# Supplementary material for: Effect of Tremella fuciformis and Different Hydrocolloids on the Quality Characteristics of Wheat Noodles
Source: Foods. 2022 Aug 29;11(17):2617. doi: 10.3390/foods11172617 (PMC9455474; doi:10.3390/foods11172617)
Supplement: Supplementary file 1 [file foods-11-02617-s001.zip › foods-1877190-Supplementary materials.pdf]

Methods

Method 1: Steady shear measurement and dynamic viscoelastic behavior of TF solution

All samples were determined by a rheometer (MARS600, Thermo Fisher, USA) equipped with a cone plate (30 mm diameter). For steady tests, samples were sheared at a shear rate ranging from 0.1 to 100 s^{-1}. Effects of shear rate on shear stress of different concentration TF solutions (80 mg/mL, 90 mg/mL, 100 mg/mL) were also determined at this condition, and the flow behavior was analyzed using the power-law model:

$$\tau = K\gamma^n \qquad (1)$$

where τ, K, γ, and n are shear stress (Pa), consistency coefficient ($Pa{\cdot}s^n$), shear rate (s^{-1}), and flow behavior index, respectively.

The linear viscoelastic regions of different concentration TF solutions (80 mg/mL, 90 mg/mL, 100 mg/mL) were analyzed by amplitude scanning mode. Their storage modulus (G') and loss modulus (G'') was measured at a shear rate of 50 s^{-1}, the strain of 5% and angular frequency from 0.1 to 100 rad/s.

Method 2: Tensile strength

Tensile strength of the different formula noodles were performed by a texture analyzer (TA-XT Plus, Stable Micro Systems, UK). The fresh noodles were boiled to the optimum cooking time and rinsed with cold water for 20 s, one drained noodle was placed on the special stage equipped with the texture analyzer, and used testing the A/KIE probe at a speed of 3.30 mm/sec. Three parallel experiments were conducted for each sample. Parameter setting: Pre-test speed: 2.00 mm/s, Test speed: 1.00 mm/s, Post-speed speed: 10.00 mm/s, Test distance: 20.00 mm.

Method 3: Intermolecular force of protein

Prepared Tris HCl buffer with pH = 8 and 30 mM, prepared reagent containing 1% SDS to determine hydrophobic interaction, reagent containing 6 mol/L urea to determine hydrogen bond, and reagent containing 1% SDS and 6 mol/L urea to determine hydrophobic interaction and hydrogen bond. 0.3 g samples (Blank, 3%TF, 0.6% SA, 0.4%GG and 0.4% XG noodle samples) were weighed and dissolved in 5 ml of the above solution, centrifuge at 4000 r/min for 10 min, 2 ml supernatant was mixed with 2 ml of 20% (w/v) trichloroacetic acid for 30 min, centrifuge at 4000 r/min for 10min, dissolve the precipitation with 0.5 mol/L NaOH, determine

the protein content by biuret method, and determine the absorbance value at 540 nm.

Method 4: Freezable water and non-freezable water content

A DSC device (Q-200, TA Instruments, USA) was used to analyze the moisture morphology of noodles. Different samples (10 mg) were obtained by using a blade and then sealed in aluminum pans. The samples were held at -20 °C for 10 min for the equilibrium of temperature and then from -20 °C to 40 °C at a rate of 5 °C/min. An empty pan was used as a reference. Nitrogen was used as a carrier gas at a 20 mL/min flow rate, and the enthalpy (ΔHw) was measured. Different noodle samples were placed in sealed bags at 25 °C for 0 h, 4 h and 8 h respectively, then repeated. The freezable water (FW) and non-freezable water (NFW) content were calculated based on Eq. (2), (3).

$$\text{FW\%} = \frac{\Delta Hw}{\Delta H0} \times 100\% \qquad (2)$$

$$\text{NFW} = \text{TW-FW} \qquad (3)$$

Where ΔHw indicated the enthalpy change per unit mass calculated from the heat absorption peak area. ΔH0 was the enthalpy of water in the dough (334 J/g), and TW indicated the total moisture content in the samples.

Method 5: Antioxidant activity

Preparation of noodle polyphenol extract: 2.5 g of freeze-dried cooked noodles were weighed and mixed with 50 mL of 80% methanol solution, incubated at 37 °C with a stirring rate of 190 rpm for 2 h, sonicate for 30 min, centrifuged at 6000 r/min for 20 min, took the supernatant and set aside.

Noodle polysaccharide extraction: 30 g of freeze-dried cooked noodles were weighed and mixed with anhydrous ethanol, incubated at 70 °C for 2 h, repeated 2 times, the residue was collected and boiled in distilled water for 3 h at 90 °C, repeated 2 times. After filtration to remove debris fragments, the filtrate was concentrated in a rotary evaporator. Protein was removed with the Sevag method. Then the solution was precipitated with four volumes of anhydrous ethanol for 24 h at 4 °C. Polysaccharide samples were obtained after freeze-drying.

Determination of DPPH radical scavenging ability: Different volumes of polyphenol extracts (2 mL of polysaccharides of different mass concentrations) were mixed with 2 mL of DPPH solution (50 μM), and the total volume was maintained at 4 mL with anhydrous ethanol.

Absorbance values were measured at 517 nm after 1 h of reaction protected from light. The calculation formula was shown in Eq. (4):

$$\text{DPPH radical scavenging rate (\%)} = \left(1 - \frac{A_{sample} - A_{background\ control}}{A_{blank}}\right) \times 100\% \quad (4)$$

Where A_{sample}, $A_{background\ control}$ and A_{blank} represent the absorbance values of the sample, background control (ethanol solution instead of DPPH solution) and blank control (distilled water instead of the sample), respectively. The volume number of polyphenol extracts (polysaccharide mass concentration) at 50% radical scavenging was calculated according to the above equation, which is the semi-inhibition rate (IC_{50}), as below.

Determination of ABTS radical scavenging ability: 0.0384 g of ABTS to 10 mL and 0.0134 g of potassium persulphate were weighed and mixed evenly to 10 mL, kept away from light for 12 h to obtain ABTS stock solution. The ABTS stock solution was diluted with 95% ethanol to an absorbance of 0.70 ± 0.02 (734 nm). Different volumes of polyphenol extracts (1 mL of polysaccharides of different mass concentrations) were mixed with 5 mL of ABTS reagent, added distilled water to make a total volume of 6 mL, avoid light reaction for 20 min, measured the absorbance at 734 nm. ABTS radical scavenging rate was calculated as in Eq. (5):

$$\text{ABTS radical scavenging rate (\%)} = \left(1 - \frac{A_{sample}}{A_{blank}}\right) \times 100\% \quad (5)$$

Where A_{blank} was the blank control absorbance value(distilled water instead of sample solution), A_{sample} was the sample solution absorbance value.

Trolox was the standard and the DPPH and ABTS clearance of the polyphenol extracts were expressed as Trolox equivalent (mg/mL).

Figure Captions

Figure S1: The shear viscosity (a) and shear stress (b) of TF solutions varied with the shear rate.

Figure S2: Shear storage modulus G′ and loss modulus G′′ of different concentration TF solutions.

Figure S3: Effect of TF, SA, GG and XG on the tensile properties of noodles (a: noodles soaked for 0 min after cooking, b: noodles soaked for 5 min after cooking, c: noodles soaked for 10 min after cooking). Values with the same superscript letters in in different figures are not significantly different at $p < 0.05$.

Figure S4: The T_2 distribution of relaxation times of TF, SA, GG and XG noodles placed at 0 h.

Figure S1

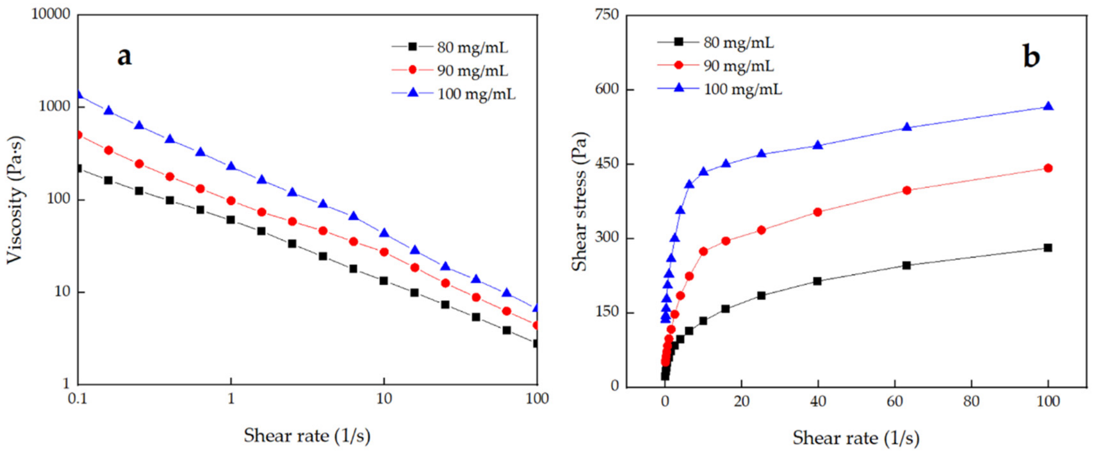

a
b
80 mg/mL
90 mg/mL
100 mg/mL
Viscosity (Pa·s)
Shear stress (Pa)
Shear rate (1/s)
Shear rate (1/s)

Figure S2

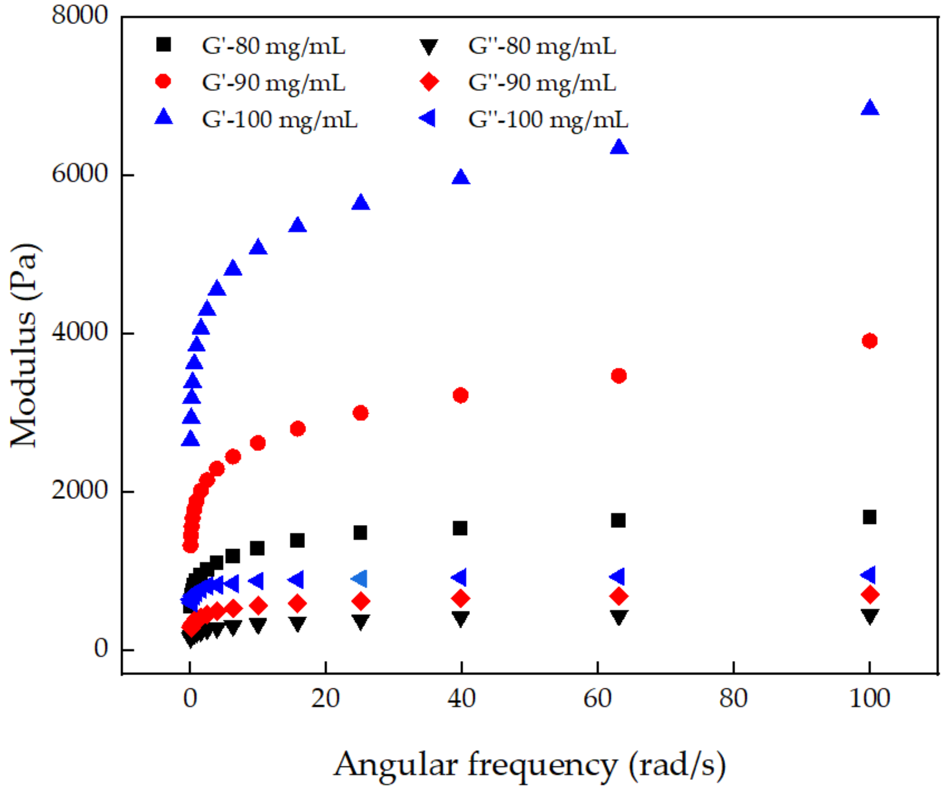
G'-80 mg/mL
G''-80 mg/mL
G'-90 mg/mL
G''-90 mg/mL
G'-100 mg/mL
G''-100 mg/mL
Modulus (Pa)
0
2000
4000
6000
8000
0
20
40
60
80
100
Angular frequency (rad/s)

Figure S3

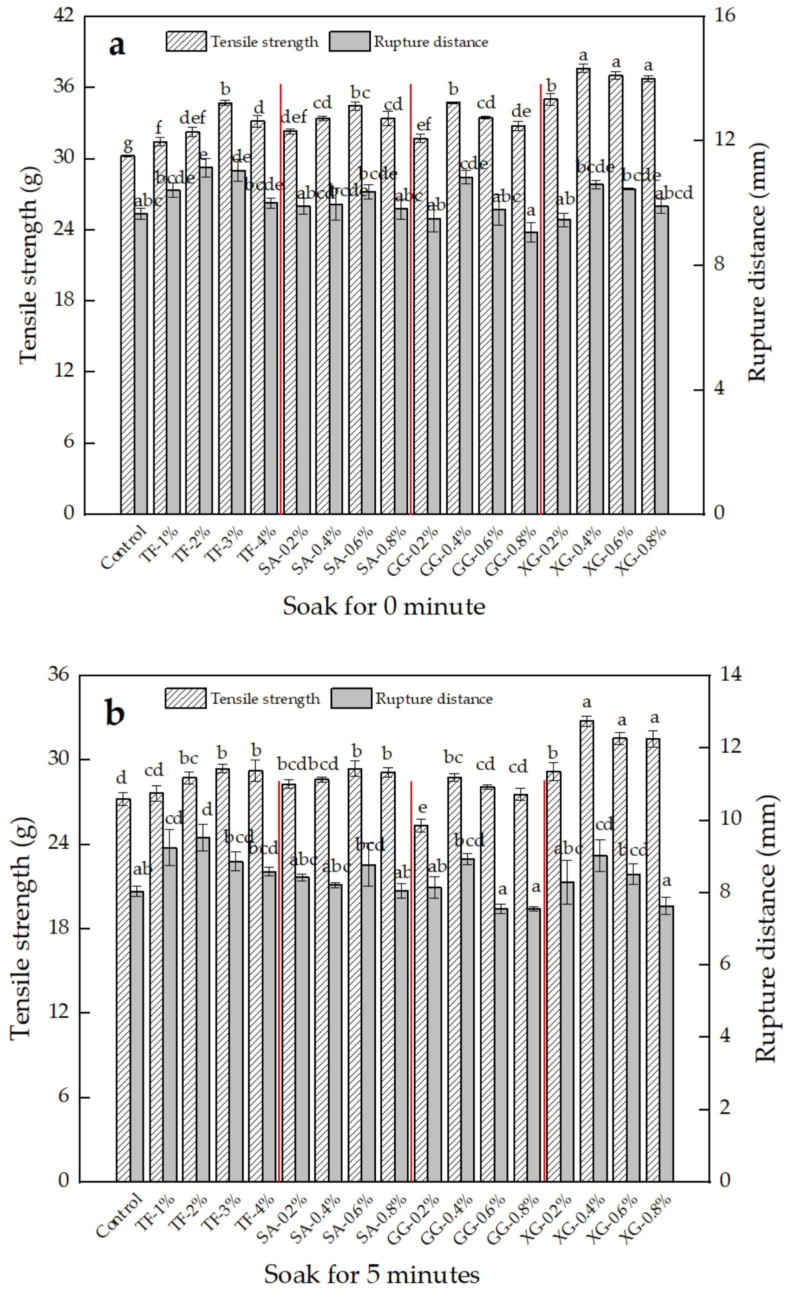

a
Tensile strength
Rupture distance
Tensile strength (g)
Rupture distance (mm)
Control
TF-1%
TF-2%
TF-3%
TF-4%
SA-0.2%
SA-0.4%
SA-0.6%
SA-0.8%
GG-0.2%
GG-0.4%
GG-0.6%
GG-0.8%
XG-0.2%
XG-0.4%
XG-0.6%
XG-0.8%
Soak for 0 minute
b
Tensile strength
Rupture distance
Tensile strength (g)
Rupture distance (mm)
Soak for 5 minutes

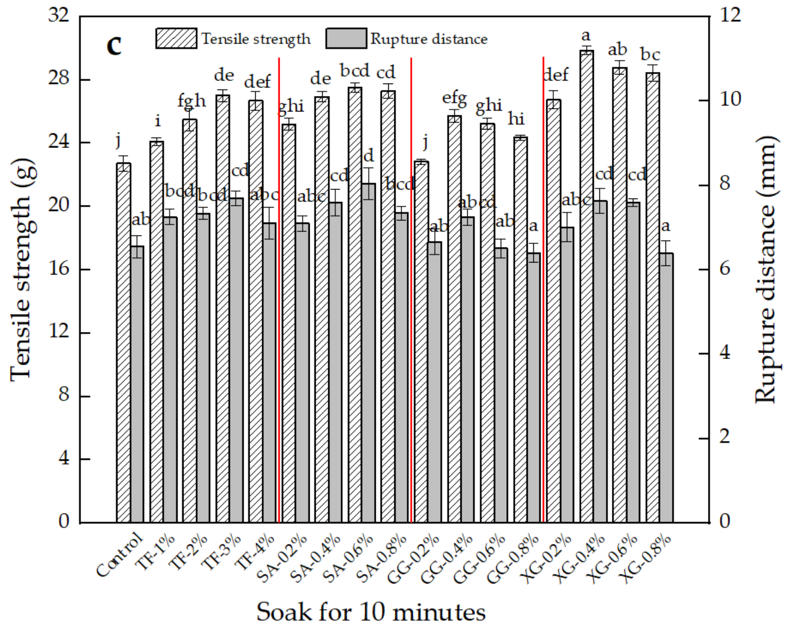
c
Tensile strength
Rupture distance
Tensile strength (g)
Rupture distance (mm)
Control
TF-1%
TF-2%
TF-3%
TF-4%
SA-0.2%
SA-0.4%
SA-0.6%
SA-0.8%
GG-0.2%
GG-0.4%
GG-0.6%
GG-0.8%
XG-0.2%
XG-0.4%
XG-0.6%
XG-0.8%
Soak for 10 minutes

Figure S4

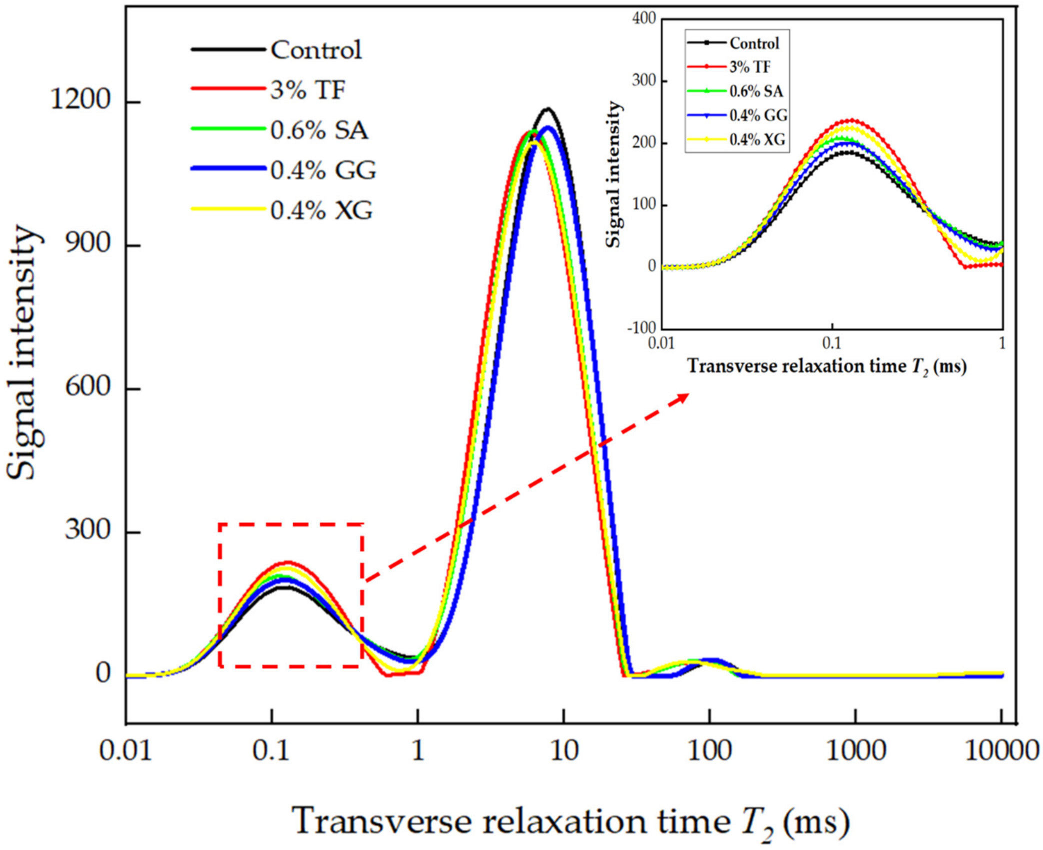

Control
3% TF
0.6% SA
0.4% GG
0.4% XG
Signal intensity
Transverse relaxation time T_2 (ms)

Table Captains

Table S1: Different concentration of TF solutions fitting the powder model $\tau = k\gamma^n$.

Note: In this table, τ, K, γ, and n are shear stress, consistency coefficient, shear rate, and flow behavior index, respectively.

Table S2: Effect of TF, SA, GG and XG on the intermolecular force of proteins. Values with the same superscript letters in a column are not significantly different at $p < 0.05$.

Table S3: Changes of moisture content of TF, SA, GG and XG noodles under different storage time. Values with the same superscript letters in a column are not significantly different at $p < 0.05$.

Table S4: Effect of TF and SA, GG, XG on DPPH and ABTS free radical scavenging rate of noodles.

Table S1

Concentration (mg/mL)	k	n	R^2
80	57.361	0.421	0.999
90	96.845	0.292	0.997
100	220.478	0.214	0.999

Table S2

Sample	Hydrophobic interaction /(mg/mL)	Hydrogen bond /(mg/mL)	Hydrophobic+ Hydrogen bond /（mg/mL）
Control	1.24±0.02[a]	1.29±0.01[d]	1.24±0.01[d]
3% TF	1.05±0.01[bc]	1.44±0.03[bc]	1.35±0.01[bc]
0.6% SA	1.03±0.02[cd]	1.47±0.01[ab]	1.37±0.00[b]
0.4% GG	1.08±0.02[b]	1.42±0.01[c]	1.32±0.02[c]
0.4% XG	0.98±0.01[d]	1.52±0.01[a]	1.42±0.01[a]

Table S3

Treatment	Sample	ΔHw /J·g^{-1}	%	FW/%	%	NFW/%	%
0 h	Control	45.16±0.10^{a}	/	13.52±0.11^{a}	/	20.33±0.11^{e}	/
	3% TF	40.21±0.13^{d}	/	12.04±0.10^{cd}	/	22.26±0.10^{bc}	/
	0.6% SA	40.35±0.21^{cd}	/	12.08±0.11^{bcd}	/	22.35±0.11^{abc}	/
	0.4% GG	40.61±0.18^{b}	/	12.16±0.07^{bc}	/	21.82±0.07^{d}	/
	0.4% XG	40.41±0.28^{bc}	/	12.10±0.08^{bc}	/	22.54±0.08^{ab}	/
4 h	Control	46.66±0.12^{a}	3.32	13.97±0.11^{a}	3.33	19.58±0.11^{d}	3.69
	3% TF	41.02±0.19^{c}	2.01	12.28±0.06^{b}	1.99	21.91±0.06^{b}	1.57
	0.6% SA	41.05±0.37^{c}	1.73	12.29±0.09^{b}	1.74	22.03±0.09^{ab}	1.43
	0.4% GG	41.58±0.07^{b}	2.39	12.45±0.15^{b}	2.38	21.42±0.15^{c}	1.83
	0.4% XG	41.25±0.05^{bc}	2.79	12.35±0.07^{b}	2.07	22.19±0.07^{a}	1.55
8 h	Control	47.46±0.47^{a}	5.09	14.21±0.11^{a}	5.10	19.07±0.11^{c}	6.20
	3% TF	41.42±0.19^{c}	3.01	12.40±0.03^{c}	2.99	21.51±0.03^{b}	3.37
	0.6% SA	41.48±0.23^{c}	2.80	12.42±0.04^{bc}	2.81	21.64±0.04^{ab}	3.18
	0.4% GG	42.05±0.05^{b}	3.55	12.59±0.05^{b}	3.54	21.01±0.05^{c}	3.71
	0.4% XG	41.72±0.036^{bc}	3.24	12.49±0.12^{bc}	3.22	21.88±0.12^{a}	2.92

Table S4

	IC_{50}/ (μg/mL)	Polyphenol-IC_{50} equivalent/ (μg/mL)		Polysaccharide- IC_{50}/ (μg/mL)	
	Vc	TF	Control/SA/GG/XG	TF	Control/SA/GG/XG
DPPH radical scavenging rate	22.59	75.30	N.D.	70.42	N.D.
ABTS radical scavenging rate	6.91	43.19	N.D.	53.79	N.D.
